# Effect of pre-operative radiation therapy on surgical outcome in retroperitoneal sarcoma

**DOI:** 10.3389/fsurg.2023.1209698

**Published:** 2023-06-12

**Authors:** Sung Jun Jo, Sean S. W. Park, Gyu Sang Yoo, Kyeong Deok Kim, So Hee Lim, Jinseob Kim, Min Jung Kim, Jeong Il Yu, Jae Berm Park, Kyo Won Lee

**Affiliations:** ^1^ Department of Surgery, Samsung Medical Center, Sungkyunkwan University School of Medicine, Seoul, Republic of Korea; ^2^ Department of Radiation Oncology, Samsung Medical Center, Sungkyunkwan University School of Medicine, Seoul, Republic of Korea; ^3^ Department of Surgery, Inha University Hospital, Inha University School of Medicine, Incheon, Republic of Korea; ^4^ Department of Epidemiology, School of Public Health, Seoul National University, Seoul, Republic of Korea; ^5^ Department of Surgery, Seoul Medical Center, Seoul, Republic of Korea

**Keywords:** retroperitoneal sarcoma, preoperative radiation therapy, saftey and effectiveness, morbididty, surgical outcome

## Abstract

**Background:**

A high rate of locoregional recurrence is one of the major difficulties in successful treatment of retroperitoneal sarcoma (RPS). Although pre-operative radiation therapy (RT) is considered a potential way to improve local recurrence, concerns about the associated treatment toxicity and risk of peri-operative complications need to be addressed. Hence, this study investigates the safety of pre-operative RT (preRTx) for RPS.

**Methods:**

A cohort of 198 patients with RPS who had undergone both surgery and RT was analyzed for peri-operative complications. They were divided into three groups according to the RT scheme: (1) preRTx group, (2) post-operative RT without tissue expander, and (3) post-operative RT with tissue expander.

**Results:**

The preRTx was overall well tolerated and did not affect the R2 resection rate, operative time, and severe post-operative complications. However, the preRTx group was associated with higher incidence of post-operative transfusion and admission to intensive care unit (*p* = 0.013 and *p* = 0.036, respectively), where preRTx was an independent risk factor only for the post-operative transfusion (*p* = 0.009) in multivariate analysis. The median radiation dose was the highest in preRTx group, although no significant difference was demonstrated in overall survival and local recurrence rate.

**Conclusion:**

This study suggests that the preRTx does not add significant post-operative morbidity to the patients with RPS. In addition, radiation dose elevation is achievable with the pre-operative RT. However, a meticulous intra-operative bleeding control is recommended in those patients, and further high-quality trials are warranted to evaluate the long-term oncological outcomes.

## Introduction

Soft tissue sarcomas are uncommon malignancy, comprising approximately 1% of all solid malignancies (1). Between 15% and 20% of them originate from the retroperitoneal space, representing a rare tumor of heterogenous histological subtypes. The backbone of curative treatment is *en bloc* resection of the primary tumor (2). However, complete resection with adequate microscopic margin is difficult or even impossible at times, due not only to the confined anatomic characteristics of the retroperitoneum, but also to the proximity of the adjacent vital structures (2–4). Consequently, the rate of complete resection, which is the most dominant predictor of long-term survival outside of tumor biology, is only achieved in 40%–60% of cases (5, 6). Unfortunately, even in completely resected retroperitoneal sarcomas (RPS), the rate of locoregional recurrence is unacceptably high, occurring in up to 50% of cases. This has been a major barrier to successful management of RPS, with five-year survival for all subtypes being about 60% at best (3, 7).

In an attempt to resolve this issue and obtain better local control of the disease, multimodal treatment approach involving radiation therapy and/or chemotherapy has been endeavored, but concrete evidence for their benefit is currently lacking (8). Chemotherapy has minimal effect, and RT to the retroperitoneum is complex with potential adverse effects to the surrounding vital organs (9). Various publications, largely of small retrospective studies, have demonstrated potential roles of pre-operative RT in improving local control and survival in RPS, including better defined target volume with more oxygenated tumor cells, reduced tumor seeding, improved tumor resectability, and minimization of unnecessary irradiation to adjacent radiosensitive tissues, especially small bowel, and thus better tolerability of RT at higher dose (8, 10, 11). Moreover, a review by Diamantis et al. suggested a potential survival benefit from peri-operative RT in RPS (12). Recently, The STRASS trial, the first and only randomized multicenter study to date, has been published. Unfortunately, however, the trial failed to demonstrate the role of pre-operative RT in improving recurrence free survival as well as overall survival in retroperitoneal sarcoma, except for the liposarcoma group in an unplanned subgroup analysis (13).

Nonetheless, RT has been increasingly utilized for RPS over the past decade, influenced largely by its established role in extremity sarcoma. Its use is still center-dependent, and more high-quality randomized controlled trials are warranted to reach a consensus and form a treatment guideline (8, 14). With the uncertainty about the long-term oncologic benefits of neoadjuvant RT, a significant concern has been raised about the treatment toxicity from the radiation and associated peri-operative morbidity by making the operation difficult. This has contributed to limiting widespread adoption of pre-operative RT (10, 15). Despite the general view that the pre-operative RT can be safely administered to the retroperitoneum, there are limited data on the short-term post-operative outcomes (6, 16). Therefore, we aim to investigate the contribution of pre-operative RT to the short-term surgical morbidity associated with RPS resection as well as its effect on survival.

## Materials and methods

### Ethics approval

This study was approved by the Samsung Medical Center Institutional Review Board (SMC IRB 2022-08-135). The need for informed consent was waivered by the board as the study did not involve any patient contact.

### Inclusion and exclusion criteria

This retrospective study included adult patients who underwent both RT and surgery for retroperitoneal sarcoma between October 2001 and February 2020 at Samsung Medical Center, Seoul, Korea. Patients were excluded if they received neither pre-operative RT nor post-operative RT. Patients treated with palliative intent were also excluded from the study.

### Patients

A cohort of 198 patients was reviewed and analyzed. The diagnosis of sarcoma and its subtypes were confirmed by reviewing the final histopathology report of the resected specimen. We then searched their medical records to confirm that those patients received peri-operative radiation therapy. They were then divided into three groups according to the RT scheme: Group (1) pre-operative RT group (preRTx), Group (2) post-operative RT without tissue expander group (postRTx), and Group (3) post-operative RT with tissue expander group (postRTx + TE). The tissue expander insertion was intraoperatively inserted at the SMC to overcome the radiation vulnerability of surrounding normal organs and deliver optimal RT doses post-operatively (3, 17, 18).

The decision to administer preoperative RT was made by a dedicated multidisciplinary sarcoma team. Pre-operative RT was only considered for patients after 2019, and the main consideration was local control of the posteromedial margin rather than cytoreduction or R0 resection. The decision was made based on the location of the tumor rather than the size of the tumor and the consent of patient for the pre-operative biopsy. Following the completion of pre-operative RT, patients were re-staged, and the surgery was performed on average 5 weeks later. All patients in this study underwent surgical resection with curative intent (R0 or R1), an *en bloc* resection of the tumor and involved adjacent organs without tumor fragmentation. After the surgery, follow-up surveillance with abdominopelvic and chest CT scans was performed every three months for the first two years, every six months for the next three years, and then yearly, to evaluate for locoregional and distant metastasis.

### Study outcomes

Our primary outcome measures were post-operative morbidity related to pre-operative RT detected during the follow-up period. The variables reflecting the morbidity entailed post-operative complication graded by Clavien-Dindo classification, length of hospital stay, need for transfusion and re-operation, and unexpected admission to intensive care unit (ICU). The secondary outcomes of interest entailed the rate of R2 resection, dose of radiation given and its tolerability, local recurrence, and survival. Local recurrence was defined as recurrence in the retroperitoneal space, as demonstrated on imaging, excluding distant metastasis. The final histopathology was reviewed to assess microscopic margins of the resection specimen and was subsequently classified as R0, R1, or R2.

### Radiation therapy protocol

In terms of the RT, the following protocol and dose escalation was uniformly implemented. Simulation computed tomography (CT) scans using contrast agent were performed for all patients. Patients were positioned supine with both arms raised and vac-lok system was used during the simulation. CT scans were obtained with slice thickness of 2.5 mm and were registered in Pinnacle (Philips, Madison, WI, USA) system to delineate the target volume. The delineated clinical target volume (CTV) included the area expanded from gross tumor volume (GTV)—the gross tumor on simulation CT or other diagnostic images—with 5–10 mm and additional subclinical disease extent decided by radiation oncologists. The planning target volume (PTV) was generated as low-risk PTV by expansion of CTV with 5–10 mm and high-risk PTV by extraction from the low-risk PTV by the volume expanded from bowel with 10 mm. The simultaneous intensity boost was applied to prescribe 62.5–70 Gy in 25 fractions to high-risk PTV and 55.0 Gy in 25 fractions to low-risk PTV.

The risk-adapted RT planning was achieved by reducing the CTV accordingly based on the proximity of adjacent radiosensitive organs such as bowel, muscle, and bony structures. The Accuray Precision™ and Pinnacle (Philips, Madison, WI, USA) were used for the RT planning of helical Tomotherapy and volumetric modulated arc therapy, respectively. Image-guided RT was performed for every session of RT using mega-voltage CT or cone-beam CT in the treatment room.

### Statistical analysis

Descriptive statistics were examined. Univariate analysis was performed using the *t*-test for continuous variables and the chi square or Fisher's exact test for categorical data, as appropriate. Multivariate odds ratios and 95% confidence intervals were calculated using logistic regression to estimate the relative odds of post-operative morbidity and outcomes by radiation therapy and the factors identified as significantly associated in the univariate analysis.

Local recurrence free survival (LRFS) and overall survival (OS) were measured from the date of surgical resection to the date of event detection or date of last follow-up visit. The Kaplan-Meier method was used to estimate survival rates. All statistical analyses were conducted using the R version 4.0.4 software program (R Foundation for Statistical Computing, Vienna, Austria) and a *p*-value of <0.05 was considered statistically significant.

## Results

Of 198 patients, pre-operative RT was performed in 23 patients (11.6%), while the remainder of the patients received post-operative RT with or without tissue expander.

### Patient demographics

When comparing the three groups based on the mode of RT, the mean age in the pre-operative RT group was the highest (64.5 ± 12.2 years), which was statistically significant (*p* = 0.008). Two patients in the preRTx group and six patients in the postRTx group received pre-operative chemotherapy, while the tissue expander group did not have anyone who received chemotherapy. Patients receiving pre-operative RT were more likely to have a pre-operative albumin <3 g/dl. There was no other significant difference in the patient characteristics including male-to-female ratio, BMI, underlying disease, as illustrated in Table 1. There was no patient with chronic obstructive pulmonary disease.

**Table 1:** Patient characteristics.

	Group 1 preRTx (*n* = 23)	Group 2 postRTx (*n* = 89)	Group 3 postRTx + TE (*n* = 86)	*p*-value
Tumor type				
Primary tumor (%)	16 (69.6)	63 (70.8)	63 (73.3)	0.909
Recurrent tumor (%)	7 (30.4)	26 (29.2)	23 (26.7)	
Age (years)	64.49 ± 12.20	55.67 ± 11.78	55.70 ± 12.65	0.008
Sex = M (%)	15 (65.2)	45 (51.1)	37 (43.0)	0.149
BMI	22.73 ± 3.04	23.58 ± 3.08	23.10 ± 2.81	0.393
BMI category (%)				0.169
<18.5	3 (13.0)	4 (4.5)	1 (1.2)	
<25	15 (65.2)	59 (67.0)	67 (77.9)	
<30	5 (21.7)	24 (27.3)	17 (19.8)	
≥30	0 (0.0)	1 (1.1)	1 (1.2)	
DM = Yes (%)	5 (21.7)	6 (6.7)	9 (10.5)	0.095
HTN = Yes (%)	10 (43.5)	24 (27.0)	27 (31.4)	0.307
Previous Abdominal surgery = Yes (%)	6 (26.1)	25 (28.1)	23 (26.7)	0.971
Pre-operative chemotherapy = Yes (%)	2 (8.7)	6 (6.7)	0 (0.0)	0.02
Hb below 10 g/dl = Yes (%)	5 (21.7)	9 (10.1)	10 (11.6)	0.302
Albumin below 3 g/dl = Yes (%)	4 (17.4)	2 (2.2)	3 (3.5)	0.017
PLT below 100 = Yes (%)	0 (0.0)	1 (1.1)	1 (1.2)	1
Follow up (days)	624.26 ± 358.44	1,360.58 ± 979.82	1,705.93 ± 1,031.74	<0.001

### Histopathology

The predominant final histopathology was liposarcoma across all groups, as shown in Table 2. There was no significant difference in tumor grade between the groups (*p* = 0.133), and Grades I–III were all present in each group. Since the significance of microscopic margin is unclear despite some evidence for better outcomes with R0 than with R1 resections (19), we have selected the rate of R2 resection as a meaningful marker of resection margin. R0/1 resection was achieved in more than 85% of the cases in all groups, and the rate of R2 resection showed no statistically significant difference between the groups. Intraoperatively, the most frequently resected adjacent organs were kidney, followed by large bowel and spleen, and there was no statistically significant difference in the number of resected organs between the groups (*p* = 0.949). In terms of the operative time, estimated blood loss and intra-operative transfusion requirement, there was no statistically significant difference.

**Table 2:** Tumor and treatment characteristics.

	Group 1 preRTx (*n* = 23)	Group 2 postRTx (*n* = 89)	Group 3 postRTx + TE (*n* = 86)	*p-*value
Tumor size (mm)	209.13 ± 111.89	147.20 ± 103.07	189.16 ± 116.51	0.013
Liposarcoma = Yes (%)	22 (95.7)	59 (66.3)	73 (84.9)	0.001
FNCLCC grade (%)				0.133
Grade I	5 (22.7)	26 (29.5)	23 (27.7)	
Grade II	10 (45.5)	28 (31.8)	41 (49.4)	
Grade III	7 (31.8)	34 (38.6)	19 (22.9)	
Resection = R2 (%)	1 (4.3)	12 (13.5)	11 (12.8)	0.557
Number of resected organ [median (IQR)]	1.00 [1.00, 2.00]	1.00 [0.00, 2.00]	1.00 [1.00, 2.00]	0.949
Large bowel = Yes (%)	8 (34.8)	33 (37.1)	15 (17.6)	0.014
Kidney = Yes (%)	15 (65.2)	53 (59.6)	59 (69.4)	0.396
Spleen = Yes (%)	1 (4.3)	12 (13.5)	13 (15.1)	0.459
Pancreas = Yes (%)	1 (4.3)	10 (11.2)	12 (14.0)	0.492
Small bowel = Yes (%)	3 (13.0)	11 (12.4)	5 (5.8)	0.263
Vascular = Yes (%)	1 (4.3)	4 (4.5)	1 (1.2)	0.386
Operative time [median (IQR)]	266.00 [224.50, 340.00]	321.00 [233.00, 412.00]	323.00 [273.00, 402.00]	0.056
Intraoperative transfusion = Yes (%)	8 (34.8)	28 (31.5)	24 (27.9)	0.775
Estimated blood loss [median (IQR)]	400.00 [275.00, 850.00]	400.00 [200.00, 1,200.00]	500.00 [262.50, 800.00]	0.994
Radiotherapy (gray)	62.50 [60.00, 62.50]	54.00 [50.10, 60.00]	58.75 [54.00, 60.00]	<0.001

### Radiation therapy

The median dose of the RT delivered in the preRTx group was 62.5 Gy (range 60.0–62.50), and it was significantly higher than that of postRTX and postRTx + TE groups (*p* < 0.001). The RT was overall well tolerated in all three groups and did not require dose limitation. Only one patient in the preRTx group did not complete the RT due to intolerance.

### Peri-operative outcomes

The post-operative complications are summarized in Table 3, and they were recorded until the time of recurrence or the last follow up. The average length of hospital stay was 22 days and was comparable between the groups (*p* = 0.728). The Clavien-Dindo (CD) complication ≥3 was defined as severe post-operative complications, and three out of five patients with severe complications in the preRTx group required operative intervention under general anesthesia (CD IIIb) for wound dehiscence, diaphragmatic hernia, and anastomotic leakage of large bowel. In the postRTx + TE cohort, there were two patients with CDIIIb; one required explantation of infected TE due to peritonitis and the other underwent repair of wound dehiscence. The rate of severe complications was statistically insignificant between the pre-operative and post-operative RT groups (*p* = 0.334). In patients with severe post-operative complications, univariate analysis was performed on various patient factors, operative factors, and tumor factors for their association (Table 4). The following factors were significantly associated with severe post-operative morbidity: BMI < 18.5 (OR = 3.49, 95% CI = 0.05–0.98, *p* = 0.047), number of resected organs (OR = 1.76, 95% CI = 1.27–2.42, *p* < 0.001), and operative time (*p* = 0.024). However, in a multivariate logistic regression model including the factors significantly associated on univariate analysis, the number of resected organs was the only independent variable that was significantly associated with increased risk of major morbidity (OR = 1.59, 95% CI = 1.12–2.27, *p* = 0.01) while the tumor factors including tumor size (OR = 1, 95% CI = 1–1.01, *p* = 0.057), FNCLCC Grade III (OR = 1.05, 95% CI = 0.43–2.59, *p* = 0.916) and liposarcoma subtype (OR = 2.28, 95% CI = 0.65–0.80, *p* = 0.199) did not. Moreover, the use of pre-operative RT did not impact on the post-operative complication (OR = 2.15, 95% CI = 0.72–6.43, *p* = 0.17).

**Table 3:** Post-operative complications.

	Group 1 preRTx (*n* = 23)	Group 2 postRTx (*n* = 89)	Group 3 postRTx + TE (*n* = 86)	*p*-value
Length of hospital stay (days)	21.78 ± 11.94	21.70 ± 15.38	20.33 ± 10.05	0.728
Post-operative transfusion = Yes (%)	7 (30.4)	7 (7.9)	7 (8.1)	0.013
Clavien-Dindo complication ≥3 (%)	5 (21.7)	11 (12.4)	9 (10.5)	0.334
Unplanned ICU Admission = Yes (%)	2 (8.7)	1 (1.1)	0 (0.0)	0.036
Need for re-operation = Yes (%)	3 (13.0)	4 (4.5)	8 (9.3)	0.215
Mortality within 30 days	0 (0)	0 (0)	0 (0)	

**Table 4:** Risk factor analysis for severe post-operative complications.

	Univariate analysis		Multivariate analysis	
	Odds ratio (95% CI)	*p*-value	Odds Ratio (95% CI)	*p*-value
Age	1.03 (1, 1.07)	0.065		
BMI < 18.5	4.55 (1.02, 20.39)	0.047	3.49 (0.7, 17.4)	0.127
DM	0.75 (0.16, 3.44)	0.71		
Hb < 10 g/dl	1.46 (0.45, 4.68)	0.527		
PLT < 100 K	7.17 (0.43, 118.39)	0.169		
Albumin <3 g/dl	2.06 (0.4, 10.53)	0.384		
Previous abdominal operation	0.63 (0.22, 1.78)	0.386		
Pre-operative radiotherapy	2.15 (0.72, 6.43)	0.17	2.61 (0.78, 8.76)	0.12
Pre-operative chemotherapy	0.99 (0.12, 8.39)	0.991		
Tumor size	1 (1, 1.01)	0.057		
Liposarcoma	2.28 (0.65, 8)	0.199		
FNCLCC Grade III	1.05 (0.43, 2.59)	0.916		
Number of resected organs	1.76 (1.27, 2.42)	<0.001	1.59 (1.12, 2.27)	0.01
Operative time	1 (1, 1)	0.024	1 (1, 1)	0.089

The need for post-operative transfusion and unplanned ICU admission were significantly higher in the preRTx group (*p* = 0.013 and *p* = 0.036, respectively). Subsequently in the multivariate analysis, albumin <3 g/dl (*p* = 0.029) was the only significant independent risk factor for the post-operative ICU admission while the pre-operative RT (*p* = 0.242) did not demonstrate a meaningful association (Supplementary Table S1). On the other hand, the need for post-operative transfusion was significantly associated with the use of pre-operative RT in both univariate and multivariate analysis (*p* = 0.002, *p* = 0.009, respectively) (Supplementary Table S2). Despite these differences, there was no statistically significant difference in mortality between all groups.

### Survival and local recurrence rate

**Figure 1:**
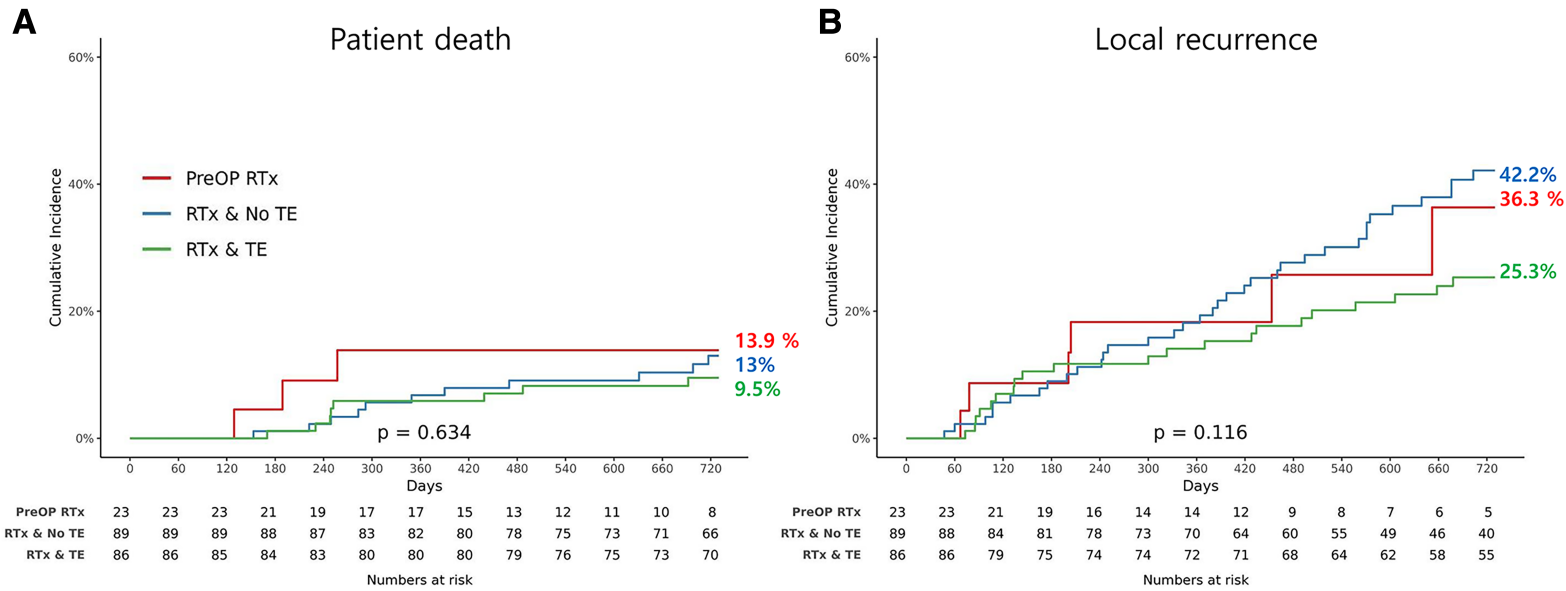
Kaplan-Meier graph for (**A**) overall survival and (**B**) incidence of local recurrence for each group.

The OS and LRFS rates are illustrated in Figure 1. The OS until the last follow up was compared between the three groups, and there was no significant difference (*p* = 0.634). In addition, there was no evidence of statistically significant difference in LRFS between the groups (*p* = 0.116). However, the patients in the preRTx cohort had a shorter follow-up duration with an average of 625 days, as compared to the other two groups: 1,361 days for postRTx, and 1,706 days for postRTx + TE group.

## Discussion

Most of the evidence for neoadjuvant RT in sarcoma are derived from multiple randomized trials in extremity sarcoma (20). However, at the same time, it is well recognized that those patients are at increased risk of post-operative complications including poor wound healing and surgical site infections, as shown in a randomized trial, where the rate of wound complication was twice as common in the neoadjuvant group (35%) when compared to the adjuvant radiation cohort (17%) (21). Using the database at Samsung Medical Center, we have found that the patients in the neoadjuvant group for retroperitoneal sarcoma were older and the primary tumor was significantly larger, consisting largely of G2 liposarcoma. In contrast to concerns about adverse effects of RT, the use of pre-operative RT did not demonstrate any significant impact on the operative time, the length of hospital stay, the need for intra-operative transfusion and re-operation, and severe post-operative complications defined as CD ≥3. The rate of concomitant adjacent organ resection was similar between the groups. However, a statistically significant increase was observed in the need for post-operative transfusion and unplanned admission to ICU in the preRTx patients. The age and tumor size did not show any significant association, but pre-operative RT was an independent risk factor for the post-operative transfusion (*p* = 0.009). Therefore, we recommend that a meticulous bleeding control is accomplished during operation in those patients. However, multivariable analysis revealed that preoperative RT was not independent risk factor for severe post-operative complication. Therefore, these findings suggest that the use of pre-operative RT seems to be safe, and this is in keeping with previous analysis from NSQIP data. Nussbaum et al. investigated a total of 785 patients undergoing RPS resection, where 71 patients (9%) received pre-operative RT and reported that the pre-operative RT did not increase 30-day morbidity or mortality (7). Bartlett et al. also used the NSQIP data, analyzing 696 patients where 70 patients (10%) received pre-operative RT, and reported similar findings (16).

In terms of the radiation therapy, previous studies have reported that RT dose escalation resulted in improved local control, tumor response, and even cancer-specific survival in various solid tumors (22–26). With the advancement of RT techniques, considerable efforts have been directed towards delivery of higher dose radiation, especially in the radiation-resistant solid tumors (27). Currently suggested dose-fractionation regimen in the neoadjuvant RT for RPS is 50 Gy in 25 fractions or 50.4 Gy in 28 fractions as the guideline from American Society for Radiation Oncology recommended based on the STRASS trial (28). However, the quality of evidence for the recommendation is moderate and some reports of dose escalation with IMRT and SIB were discussed in the guideline as showing acceptable toxicity and encouraging early local control (28–31). One of the studies, by Tzeng et al., investigated the feasibility and outcomes of dose escalation in the pre-operative RT with selective dose escalation to the margin at risk for the patients with retroperitoneal sarcoma (31). In that study, 45 Gy in 25 fractions was delivered to the entire tumor bed and surrounding margin and the boost dose up to 57.5 Gy to the volume predicted as high risk for positive surgical margins. Despite the reports of tolerability of such RT regimen and high rates of tumor response and complete resection, subsequent analysis of the relevant clinical outcomes from dose escalation in neoadjuvant setting has not been conducted. Instead, intraoperative RT boost with dose escalation has been attempted for the at-risk area in addition to the neoadjuvant RT, the results of which demonstrated improved local control and overall survival (5, 32, 33). In our cohort, we have found that higher dose of radiation was possible in the pre-operative RT group with median dose of 62.5 Gy, which was even higher than that in the TE group (median dose of 58.8 Gy), and it was overall well tolerated. This is helpful as the pre-operative RT is often preferred over adjuvant RT for the protective effect from the primary retroperitoneal sarcoma on the adjacent radiosensitive organs (34).

The overall survival and local recurrence free survival did not demonstrate statistically significant difference between the three groups. However, it requires a careful interpretation as the analysis is limited by the short-term follow up period in the preRTx group, where the other two groups had 2–3 times longer follow up duration, and this difference was statistically significant (*p* < 0.001). The authors are planning to conduct subsequent analysis with longer follow up to better assess the oncological and survival benefit of the pre-operative RT. This study has other limitations to note. Most importantly, the retrospective nature of the study conducted at a single institution entails potential selection bias, and our findings may not be generalizable to other cohorts of patients. In addition, a small sample size, particularly in the pre-operative group, further limits the study, although the issue of overall small sample size is somewhat attributed to the low incidence of retroperitoneal sarcoma. Nonetheless, our study demonstrates that the addition of pre-operative RT to curative resection of retroperitoneal sarcoma does not appear to increase the peri-operative morbidity and mortality, and that it is safe and feasible.

## Conclusion

In conclusion, this study describes a single institution cohort of patients undergoing curative resection of retroperitoneal sarcoma with peri-operative RT at a dedicated sarcoma center. Despite presenting with older age and larger tumors, the use of pre-operative RT did not add any statistically significant morbidity to the peri-operative outcomes, except for the post-operative transfusion requirement. Therefore, we recommend a meticulous intra-operative bleeding control in those patients having undergone pre-operative RT. However, exaggerated concern for increased peri-operative complications should not exclude appropriately selected patients from receiving potentially valuable pre-operative RT. Further study is warranted to better define the long-term sequelae of radiation as well as its oncologic efficacy in patients with RPS.

## Data Availability

The raw data supporting the conclusions of this article will be made available by the authors, without undue reservation.
