# Supplementary material for: Effect of pre-operative radiation therapy on surgical outcome in retroperitoneal sarcoma
Source: Front Surg. 2023 Jun 12;10:1209698. doi: 10.3389/fsurg.2023.1209698 (PMC10291121; doi:10.3389/fsurg.2023.1209698)
Supplement: Supplementary file 1 [file Table1.docx]

**Supplementary Table 1** Risk factor analysis for ICU admission.

|  | Univariate Analysis | | Multivariate Analysis | |
| --- | --- | --- | --- | --- |
|  | **Odds Ratio (95% CI)** | **p value** | **Odds Ratio (95% CI)** | **p value** |
| Age | 1.14 (1,1.3) | 0.059 |  |  |
| BMI <18.5 | 13.36 (1.08,165.4) | 0.043 | 1.75 (0.06, 54.55) | 0.751 |
| DM | 4.63 (0.4,53.5) | 0.219 |  |  |
| Hb < 10 g/dl | 3.74 (0.33,42.88) | 0.289 |  |  |
| PLT < 100K | 0 (0,Inf) | 0.996 |  |  |
| Albumin < 3 g/dl | 53.71 (4.34,665.11) | 0.002 | 24.52 (1.39, 431.49) | 0.029 |
| Previous Abdominal Operation | 0 (0,Inf) | 0.994 |  |  |
| Pre-operative Radiotherapy | 16.57 (1.44,190.66) | 0.024 | 5.84 (0.3, 112.22) | 0.242 |
| Pre-Operative Chemotherapy | 0 (0,Inf) | 0.995 |  |  |
| Tumor Size | 1.01 (1,1.02) | 0.052 |  |  |
| Liposarcoma | 16977525.2 (0,Inf) | 0.995 |  |  |
| FNCLCC Grade III | 1.11 (0.1,12.49) | 0.933 |  |  |
| Number of Resected Organs | 1.25 (0.54,2.93) | 0.6 |  |  |
| Operative Time | 1 (0.99,1.01) | 0.996 |  |  |

**Supplementary Table 2** Risk factor analysis for post-operative transfusion.

|  | Univariate Analysis | | Multivariate Analysis | |
| --- | --- | --- | --- | --- |
|  | **Odds Ratio (95% CI)** | **p value** | **Odds Ratio (95% CI)** | **p value** |
| Age | 1.02 (0.99,1.06) | 0.21 |  |  |
| BMI <18.5 | 2.98 (0.56,15.83) | 0.199 |  |  |
| DM | 2.37 (0.71,7.89) | 0.161 |  |  |
| Hb < 10 g/dl | 2.6 (0.86,7.9) | 0.092 |  |  |
| PLT < 100K | 8.8 (0.53,146.19) | 0.129 |  |  |
| Albumin < 3 g/dl | 4.75 (1.09,20.63) | 0.038 | 2.9 (0.59,14.24) | 0.189 |
| Previous Abdominal Operation | 0.41 (0.12,1.46) | 0.169 |  |  |
| Pre-operative Radiotherapy | 5.03 (1.77,14.27) | 0.002 | 4.27 (1.44,12.66) | 0.009 |
| Pre-Operative Chemotherapy | 1.21 (0.14,10.38) | 0.859 |  |  |
| Tumor Size | 1 (1,1.01) | 0.111 |  |  |
| Liposarcoma | 6.42 (0.84,49.24) | 0.074 |  |  |
| FNCLCC Grade III | 1.12 (0.43,2.94) | 0.814 |  |  |
| Number of Resected Organs | 1.25 (0.88,1.77) | 0.208 |  |  |
| Operative Time | 1 (1,1) | 0.107 |  |  |

**Supplementary Table 3** Histological characteristics.

|  | **Group 1**  **preRTx (n = 23)** | **Group 2**  **postRTx (n = 89)** | **Group 3**  **postRTx+TE (n = 86)** | **p value** |
| --- | --- | --- | --- | --- |
| Histologic subtype (%) |  |  |  |  |
| Liposarcoma | 22 (95.7) | 59 (66.3) | 73 (84.9) | 0.001 |
| Leiomyosarcoma | 0 (0.0) | 20 (22.5) | 6 (7.0) |  |
| Malignant peripheral nerve sheath tumor | 0 (0.0) | 1 (1.1) | 0 (0.0) |  |
| Others | 1 (4.3) | 9 (10.1) | 7 (8.1) |  |
| Tumor differentiation (%) |  |  |  | 0.359 |
| 1 | 5 ( 21.7) | 26 ( 29.2) | 23 ( 26.7) |  |
| 2 | 0 ( 0.0) | 7 ( 7.9) | 2 ( 2.3) |  |
| 3 | 18 ( 78.3) | 56 ( 62.9) | 60 ( 69.9) |  |
| Unknown | 0 ( 0.0) | 0 ( 0.0) | 1 ( 1.1) |  |
| Necrosis (%) |  |  |  | 0.799 |
| Absent | 9 ( 39.1) | 50 ( 56.2) | 46 ( 53.5) |  |
| <50% | 9 ( 39.1) | 31 ( 34.8) | 31 ( 36.0) |  |
| >=50% | 2 ( 8.8) | 5 ( 5.6) | 5 ( 5.8) |  |
| Unknown | 3 ( 13.0) | 3 ( 3.4) | 4 ( 4.7) |  |
| Mitotic index (%) |  |  |  | 0.163 |
| <9/10 HPF | 20 ( 87.0) | 59 ( 66.3) | 65 ( 75.6) |  |
| 10-19/10 HPF | 2 ( 8.7) | 18 ( 20.2) | 11 ( 12.8) |  |
| >=20/10 HPF | 0 ( 0.0) | 10 ( 11.2) | 5 ( 5.8) |  |
| Unknown | 1 ( 4.3) | 2 ( 2.3) | 5 ( 5.8) |  |
